# The strategy of antibiotic use in critically ill neutropenic patients

**DOI:** 10.1186/2110-5820-1-22

**Published:** 2011-06-15

**Authors:** Matthieu Legrand, Adeline Max, Benoît Schlemmer, Elie Azoulay, Bertrand Gachot

**Affiliations:** 1 Department of Anesthesiology and Critical Care, Lariboisière Hospital, Assistance Publique - Hopitaux de Paris, University of Paris 7 Denis Diderot, 2 rue Ambroise-Paré, 75475 Paris, Cedex 10, France; 2 Medical Intensive Care Unit, AP-HP, Saint-Louis Hospital, 1 rue Claude Vellefaux, Assistance Publique - Hopitaux de Paris, University of Paris 7 Denis Diderot, 75010, Paris, France; 3 Department of Intensive Care and Infectious Diseases, Institut Gustave Roussy, 39, rue Camille Desmoulins, 94805 Villejuif cedex, France

## Abstract

Suspicion of sepsis in neutropenic patients requires immediate antimicrobial treatment. The initial regimen in critically ill patients should cover both Gram-positive and Gram-negative pathogens, including *Pseudomonas aeruginosa*. However, the risk of selecting multidrug-resistant pathogens should be considered when using broad-spectrum antibiotics for a prolonged period of time. The choice of the first-line empirical drugs should take into account the underlying malignancy, local bacterial ecology, clinical presentation and severity of acute illness. This review provides an up-to-date guide that will assist physicians in choosing the best strategy regarding the use of antibiotics in neutropenic patients, with a special focus on critically ill patients, based on the above-mentioned considerations and on the most recent international guidelines and literature.

## Introduction

Neutropenia is defined as a neutrophil count ≤ 500/mm^3 ^or ≤ 1000/mm^3 ^with a predicted decrease to ≤ 500/mm^3 ^[1,2]. Infection remains a major complication of neutropenia, and severe sepsis and septic shock are associated with high hospital mortality [3,4]. Fever, defined as a single oral temperature ≥38.3°C or ≥38.0°C for at least 1 hour, develops in 10-50% of patients after chemotherapy for solid tumors and in more than 80% of patients with hematological malignancies [5].

Urgent and appropriate antibiotic administration is mandatory to prevent further clinical deterioration, especially in critically ill patients with signs of respiratory distress or severe sepsis. Therefore, the first-line antibiotics should cover the pathogens deemed to be most likely based on the patient's characteristics, neutropenia, and local epidemiology. However, the changing epidemiology of infections, global increase in resistant strains, and need to contain healthcare costs require careful selection of antibiotics. Only 10-40% of episodes of febrile neutropenia are microbiologically documented in neutropenic patients, which hampers appropriate antibiotic spectrum adjustment in most cases [5]. This review provides an up-to-date guide to assist physicians in choosing the optimal antibiotic regimen in neutropenic patients, based on the above-mentioned considerations and on the most recent international guidelines and literature.

### Bacterial epidemiology in neutropenic patients

During the 1990s, Gram-positive bacteria emerged as the leading agents responsible for infections in neutropenic patients worldwide. In adults with bloodstream infections and malignancies in the United States, the proportion of Gram-positive organisms increased from 62% in 1995 to 76% in 2000, whereas the proportion of Gram-negative infections decreased from 22% to 15% [6]. Factors that may increase the risk of Gram-positive sepsis in neutropenic patients include the widespread use of central venous catheters, introduction of prophylactic quinolone therapy, increased use of proton pump inhibitors, and rising prevalence of chemotherapy-induced mucositis [7]. Importantly, Gram-negative bacteria seem to be causing an increasing number of infections in neutropenic patients since the early 2000s (Table 1). The selection of empirical antimicrobials depends in part on an assessment of which pathogens are most likely to be involved. Table 2 shows a nonexhaustive list of pathogens with their possible sites of development in neutropenic patients. Although Gram-negative bacteria are usually associated with severe infections that have high mortality rates, coagulase-negative staphylococci (CNS), which are recognized as the most common causes of nosocomial bacteremia, often are associated with more indolent forms of infections and have been more prevalent among low-risk than among high-risk patients [8]. However, in the setting of sustained bacteremia, CNS is an emerging cause of nosocomial endocarditis, usually occurring as a complication of catheter-related infection [9]. Viridans group streptococcal bacteremia may be associated with fulminant infection and is common in patients with hematological malignancies and profound neutropenia [6].

**Table 1:** Bloodstream bacterial isolates in clinical trials enrolling neutropenic adults between 1998 and 2009

	**Carratala et al. *Arch Intern Med *1998 **[46]	**Gruson et al. *Eur Respir J *1999 **[47]	**Feld et al. *J Clin Oncol *2000 **[26]	**Regazzoni et al. *Intensive Care Med *2003 **[48]	**Harter et al. Bone *Marrow Transplant *2006 **[25]	**Klastersky et al. Int J *Antimicrob Agents *2007 **[49]	**Metallidis et al**. Eur J *Intern Med *2008 [50]	**De La Rubia et al**. *Biol Blood Marrow Transplant *2009 [51]
No. of patients	39	38	411	62	161	2142	75	428
No. of organisms	43	5	93	16	96	556	13	125
**Gram-positive organisms**	**18 (41.9)**	**4 (80)**	**41 (44.1)**	**7 (43.7)**	**70 (72.9)**	**353 (63.5)**	**6 (46.1)**	**81 (64.8)**
*Staphylococcus spp *	3 (7)	3 (60)	13 (14)	2 (12.5)	52 (54.5)	187 (33.6)	6 (46.1)	56 (44.8)
*Streptococcus spp*	15 (34.9)	1 (20)	27 (29)	4 (25)	15 (15.6)	114 (20.5)	-	10 (8)
*Other*	-	-	1 (1.1)	1 (6.2)	3 (3.1)	52 (9.4)	-	15 (12)
**Gram-negative organisms**	**25 (58.1)**	**1 (20)**	**52 (55.9)**	**9 (56.3)**	**26 (27.1)**	**203 (36.5)**	**7 (53.9)**	**44 (35.2)**
*Enterobacteriaceae*	6 (14)	-	42 (45.2)	6 (37.5)	22 (23)	123 (22.1)	4 (76.9)	-
*P. aeruginosa*	17 (39.5)	1 (20)	6 (6.5)	2 (12.5)	3 (3.1)	49 (8.8)	2 (15.3)	-
*Other*	2 (4.6)	-	4 (4.3)	1 (6.3)	1 (1)	31 (5.6)	1 (7.7)	-

**Table 2:** Nonexhaustive list of bacteria that cause disease in febrile neutropenic patients, with their usual sites of development

		Site of infection
**Gram-positive bacteria**		
	Coagulase-negative staphylococci	Bloodstream infections, catheter-associated sepsis
	*Viridans *group streptococci	Bloodstream infections, endocarditis
	*Enterococcus faecium* *Enterococcus faecalis*	Bloodstream infections, endocarditis
	*Stomatococcus mucilaginosus*	Bloodstream infections, catheter-associated sepsis
	*Pediococcus species*	Urine and bloodstream infections
	*Corynebacterium jeikeium*	Endocarditis, catheter-related bacteremia, cutaneous lesions, and nodular pulmonary infiltrates
	*Lactobacillus species *	Bloodstream infections endocarditis, meningitis, intraabdominal abscesses, and pneumonia
	*Rhodococcus equi*	Suppurative pneumonia with pulmonary abscesses and empyema
	*Clostridium septicum*	Metastatic myonecrosis, typhlitis
**Gram-negative bacteria**		
	*P. aeruginosa*	Pneumonia, bloodstream infections
	*Escherichia coli*, *Klebsiella species*, *Enterobacter*	Bloodstream infections, catheter-associated sepsis, and pneumonia
	*Stenotrophomonas maltophilia*	Pneumonia, bloodstream infections
	*Alcaligenes xylosoxidans *and *Burkholderia cepacia*	Catheter-associated sepsis
	*Capnocytophaga species*	Bloodstream infections in bone marrow transplant recipients
**Anaerobes**		
	*Fusobacterium nucleatum *	Bloodstream infections, ulcerative pharyngitis, and nodular pulmonary infiltrates due to septic emboli
	*Leptotrichia buccalis*	Bloodstream infections with extensive mucosal involvement in severely immunosuppressed patients
**Mycobacteria**	*Mycobacterium chelonae* *Mycobacterium fortuitum*	Pneumonia, disseminated infections

A major concern is the emergence of multidrug-resistant bacteria [10,11]. Among Gram-negative rods, *Pseudomonas aeruginosa*, *Escherichia coli*, *Citrobacter freundii*, *Acinetobacter *species, and *Stenotrophomonas maltophilia *are increasingly found to exhibit multidrug-resistance (i.e., resistance to three or more classes of antimicrobials), extensive drug resistance (i.e., resistance to all but one or two classes), or pandrug-resistance (i.e., resistance to all available classes) [12]. Antibiotic selection pressure promotes the induction of extended-spectrum chromosomal β-lactamases (ESBL) after the use of β-lactams [13,14] and the selection of enterobacteria with decreased porin production after the use of carbapenems [11]. ESBL-producing *Enterobacteriaceae *are now commonly isolated in the community [15]. Furthermore, Enterobacteriaceae that produce *Klebsiella pneumoniae *carbapenemases (KPCs) are now reported worldwide, and KPCs have become the leading class A carbapenemases. KPC β-lactamases confer decreased susceptibility or resistance to virtually all β-lactams [16]. Similarly, fluoroquinolone exposure is associated with the emergence of methicillin-resistant *Staphylococcus aureus *(MRSA) and penicillin-resistant streptococci [17]. Widespread use of vancomycin has been described to cause outbreaks of bacteremia due to nosocomial vancomycin-resistant enterococci associated with high mortality rates [6]. Finally, other Gram-positive organisms with limited susceptibility or resistance to β-lactams have been increasingly isolated in cancer patients with febrile neutropenia; examples include *Corynebacterium jeikeum, Lactobacillus*, *Bacillus *species, and *Rhodococcus *species [12]. Antibiotic resistance rates vary widely across countries. For instance, the proportion of *P. aeruginosa *strains that exhibit carbapenem resistance is below 10% in Denmark, The Netherlands, Switzerland, Sweden, and Finland, greater than 25% in Croatia, Turkey, Germany, Italy, the Czech Republic, and as high as 45% in Greece [18].

### Principles underlying first-line antibiotic therapy

Antibiotic therapy must be initiated immediately in febrile patients with neutropenia, especially when criteria of severe sepsis are met [1,2]. The antibiotics used for first-line therapy must be active against the most likely pathogens, as estimated based on the suspected source of infection, patient's medical history, careful clinical examination, bacteriological findings, and x-ray results. Signs and symptoms of inflammation are frequently minimal or absent in patients with neutropenia. The initial assessment of patients with febrile neutropenia should include a careful physical examination for subtle signs and symptoms of infection, with special attention to the sinuses and oropharynx, skin and skin folds, intravenous lines, genital organs, and anal area. Obtaining bacterial samples is crucial to ensure the detection and susceptibility testing of the causative pathogen. Most pathogens are isolated from blood cultures, which must be drawn both from the catheter and from a peripheral vein. Cultures of stool, urine, cerebrospinal fluid, and/or skin lesions should be performed as indicated by the clinical picture. Previous microbiological results should be considered, because carriage of multiresistant strains may last several weeks or months [19]. A chest radiograph should be obtained, and high-resolution computed tomography of the chest may be indicated if the patient has pulmonary symptoms with an uninformative chest radiograph or if an invasive mold infection is suspected [1,2].

### Choice of the first-line antibiotic regimen

#### Route of empirical antibiotic therapy

Although oral antibiotic administration can be an option in neutropenic patients with the lowest risk of complications, all patients with prolonged (> 7 days duration) and/or profound neutropenia (< 100 cells/mm^3^) and/or abdominal pain, nausea and/or vomiting diarrhea and/or criteria of severe sepsis or septic shock with signs of organ failure should be treated intravenously. Suspicion of catheter-related infection or new pulmonary infiltrates are other indications of intravenous antibiotics administration.

#### Antibiotic combinations

The advantages of combination therapy include coverage of a broad spectrum of pathogens and, theoretically, synergistic activity with a decrease in the emergence of resistant strains. The main downsides are ototoxicity and nephrotoxicity, most notably with aminoglycosides, and increased cost. It should be pointed out that nephrotoxicity may occur even with very short courses of aminoglycosides, particularly with multiple-dose regimens and in patients receiving or having received other toxic substances (e.g., cisplatin, ciclosporin, amphotericin B, colistin, acyclovir, or contrast media) [20,21].

So far, no randomized study or metaanalysis has proven that adding an aminoglycoside or quinolone to a β-lactam is superior over using a broad-spectrum β-lactam alone. Results from a recent multicentric propensity matched cohort study have suggested that early combination antibiotic therapy is associated with decreased mortality in septic shock. Although not being the focus of this study, it is likely that neutropenic patients also may benefit from antibiotic combination antibiotic therapy [22]. Given that *P. aeruginosa *infection is rather common and associated with high mortality rates in neutropenic patients [23], the empirical antibiotic regimen should cover this pathogen. Monotherapy with ceftazidime, imipenem, or piperacillin/tazobactam seems to be as effective as β-lactam/aminoglycoside combinations, even in the subset of bacteremic patients [1,2].

There are no studies of β-lactam/aminoglycoside combinations in critically ill neutropenic patients. According to the aforementioned guidelines, β-lactam plus aminoglycoside combinations may be justified in patients with severe sepsis or septic shock and in those with suspected resistant Gram-negative infections. The safety of cefepime has recently been put into question by a meta-analysis, suggesting an increase risk of death associated with the use of cefepime in neutropenic patients [24]. The mechanism underlying such an association could not been identified. However, the U.S. Food and Drug Administration (FDA) still approves cefepime based on a new metaanalysis performed by the Agency, including additional data, which did not find any increase in mortality in cefepime-treated compared with control patients [2].

Ciprofloxacin has good activity against Gram-negative bacteria, including *Pseudomonas aeruginosa*, but poor coverage of Gram-positive organisms. Levofloxacin has better activity against Gram-positive organisms but less activity against *Pseudomonas aeruginosa*. However, fluoroquinolones use is associated with the emergence of antibiotic-resistant pathogens, and therefore, their use in initial empirical regimens should be discouraged, particularly in patients with a history of quinolone-based prophylaxis.

In febrile neutropenic patients, efficacy seems largely equivalent with β-lactam monotherapy by cefepime, piperacillin/tazobactam, and carbapenems [1,2,25,26] (Table 3). Ceftazidime monotherapy may be an effective strategy. However, the limited activity of ceftazidime against Gram-positive bacteria is of concern in high-risk neutropenic patients, because streptococcal bacteremia is associated with high complication rates [27]. Although most broad spectrum β-lactams (cefepime, piperacillin/tazobactam, and carbapenems) provide coverage against most Gram-positive bacteria, some Gram-positive organisms, such as *S. mitis*, methicillin-resistant *S. aureus*, *Enterococcus faecium*, and *Corynebacterium *species, may be resistant to β-lactams and susceptible only to glycopeptides (i.e., vancomycin and teicoplanin), quinupristin-dalfopristin, daptomycin, or linezolid. The appropriateness of adding vancomycin to a β-lactam has long been a matter of debate. Given the risk of emergence of resistant pathogens due to widespread vancomycin use and the often relatively indolent course of infections due to the most commonly isolated resistant Gram-positive organisms (i.e., CNS), routinely adding vancomycin to the first-line regimen is now strongly discouraged in stable patients [1,2,28]. In contrast, the vancomycin/β-lactam combination remains recommended for the first-line treatment of patients with severe sepsis or septic shock and of patients at high risk for infection by antibiotic-resistant Gram-positive cocci (i.e., those with prolonged (> 7 days) and profound (absolute neutrophil count < 100 cells/mm^3^) neutropenia and/or presenting with hypotension, pneumonia, new-onset abdominal pain, or neurologic changes [1,2], Table 3).

**Table 3:** Suggested dosages for empirical antibiotic therapy in high-risk adult neutropenic patients with normal renal function

	Dosage	Targets for serum concentrations	
**Cefepime**	2 g iv every 8-12 hours	Max. T > MIC (at least 70% of the dosing interval)	
**Piperacillin-tazobactam**^#^	4 g/500 mg iv every 6-8 hours	Max. T > MIC (at least 70% of the dosing interval)	
**Ceftazidime**^#^	1-2 g every 8 hours or 2 g loading dose followed by 6 g continuous iv infusion every 24 hours	Max. T > MIC (at least 70% of the dosing interval)	
**Imipenem**^#^	500 mg every 6 hours to 1 g iv every 6-8 hours Up to 50 mg/kg/day *for seriously ill patients: *1 g iv every 6-8 hours	Max. T > MIC (at least 70% of the dosing interval)	
**Meropenem**^#^	0.5-1 g iv infusion every 8 hours *for seriously ill patients: *1 g iv infusion every 8 hours	Max. T > MIC (at least 70% of the dosing interval)	
Amikacin*	15-20 mg/kg once daily *for seriously ill patients: 25-30 mg/kg once daily *	Peak/MIC ratio > 8-10	Peak: 64-80 μg/ml Trough < 2.5 μg/ml
Gentamicin* tobramycin*	3-5 mg/kg iv once daily *for seriously ill patients: 7-8 mg/kg iv once daily*	Peak/MIC ratio > 8-10	Peak: 32-40 μg/ml Trough < 0.5 μg/ml
Vancomycin*	15-20 mg/kg ¤ given every 8-12 hours *for seriously ill patients: loading dose of 25-30 mg/kg* or *loading dose of 15 mg/kg iv followed by 30-60 mg/kg continuous iv infusion every 24 hours*	Optimal 24 h-AUC/MIC ratio > 400	Trough: > 15-20 mg/L, 25-35 mg/L if severe infection Always > 10 mg/L to avoid the development of resistance
Teicoplanin*	6-12 mg/kg every 12 hours iv from day 1 to 4 followed by 6-12 mg/kg every 24 hours	Optimal 24 h-AUC/MIC ratio > 400	Trough: 20-30 mg/L
**Ciprofloxacin**^#^	400 mg every 8-12 hours	Optimal 24 h-AUC/MIC ratio ~125 for Gram-negative bacteria Optimal 24 h-AUC/MIC ratio ~40 for Gram-positive bacteria	
**Colimycin**	75,000-150,000 IU/kg (2.5-5 mg/kg colistin base) every 24 hours in 3 divided doses		

The local bacterial ecology and patient's bacterial history must be considered when selecting empirical antibiotics.

^#^Note that the highest suggested dosage is active against *Pseudomonas aeruginosa*.

*Routine determination of trough serum levels is required. For aminoglycosides, renal impairment may occur after a few days of treatment; therefore, treatment duration should be limited to 48-72 hours and should not exceed 5 days. Multiple daily doses of aminoglycosides are discouraged, because this regimen does not reduce toxicity and cannot provide sufficient peak serum concentrations; peak concentration should be determined 30 minutes after the end of the infusion and trough concentration just before the next infusion. For vancomycin, trough serum concentrations should be obtained just before the fourth dose. There is no evidence that continuous infusion regimens improve patient outcomes. The recommended infusion rate is 0.5-1 g/h. Monitoring of trough serum vancomycin concentrations is recommended for patients receiving aggressive dose targeting, for patients with unstable renal function, and for patients receiving concurrent treatment with nephrotoxic agents.

^¤^Should be calculated based on actual body weight.

iv, intravenous.

In a randomized, controlled study of neutropenic patients with cancer, linezolid and vancomycin produced similar outcomes [29]. The limited data on linezolid and the bacteriostatic activity of this drug are of concern when treating neutropenic patients. Linezolid may finally cause marrow suppression when given for more than 14 days. Limited data exist on daptomycin in neutropenic patients, but it may represent an alternative in selected situations [30].

In all likelihood, no guidelines of universal relevance can be developed. Instead, the global epidemiology of bacterial infections should be considered in conjunction with local rates of pathogen isolation and resistance. The patient's own ecology also should be considered. Valuable information can be garnered from a review of previous anterior nasal and rectal swabs, which may have shown colonization by antibiotic-resistant bacteria (e.g., methicillin-resistant *S. aureus, P. aeruginosa*, multidrug-resistant Gram-negative bacilli, and vancomycin-resistant enterococci). A history of infection should raise the possibility of a recurrence and should prompt the use of antibiotics active against the pathogen involved. Finally, renal and hepatic function and the risk of allergies may influence the choice of the first-line antibiotics.

### Adapting the antibiotics: treatment duration

**Figure 1:**
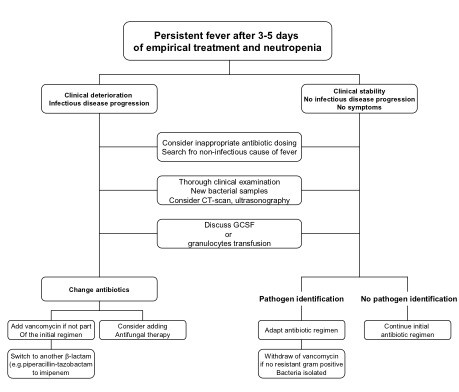
**Suggested adjustments to the empirical antibiotic regimen in patients with persistent fever after 3-5 days treatment**.

Failure to respond to empirical therapy is defined as persistent fever and the development of serious medical complications. The median time to defervescence in hospitalized patients with cancer is 5-7 days, although low-risk patients often respond within 2 days or less [1,2]. International guidelines recommend a reassessment of the initial antibiotic regimen after 3-5 days if the fever persists [1,2]. Persistence of fever with clinical deterioration or infectious disease progression should be distinguished from persistence of fever in a clinically stable patient. Patients whose clinical status deteriorates require a complete reassessment, including a careful physical examination and the collection of new culture specimens to look for a second infection. Ultrasonography and high-resolution computed tomography should be performed as indicated by the clinical data, and the indwelling catheter should be removed if a catheter-related infection is proven or strongly suspected. If a resistant pathogen is isolated or suspected to be responsible for the deterioration (i.e., if present in a recent stool culture) and is not covered by initial empirical regimen, the treatment should be modified promptly. Empirical addition of a glycopeptide, if not used initially, may be warranted, and switching from piperacillin-tazobactam or a third-generation cephalosporin to a carbapenem as second-line therapy should be considered (Figure 1). Finally, empirical addition of antifungal agents deserves consideration in patients who have risk factors for fungal infection [1,2,28,31]. The management of antifungal therapy is beyond the scope of this chapter, but readers can refer to specific reviews on this topic [31-33]. For clinically stable patients with persistent fever, there is no published evidence to support a change in the antibiotic regimen. Moreover, the widespread emergence of multiresistant and panresistant bacterial strains should discourage "escalating" strategies, such as switching from piperacillin-tazobactam or cefepime to a carbapenem or adding vancomycin. When a causative pathogen is identified, the antibiotic regimen should be adapted based on the antibiotic susceptibility test results. The dwindling number of drugs in the pharmaceutical pipeline and the increased incidence of multidrug-resistant bacteria have led to the increasing use of old antibiotics, such as colistin [34].

Colistin exhibits rapid and concentration-dependent bactericidal activity with relatively low rates of resistance. Good rates of clinical responses have been reported in patients infected with multidrug-resistant bacteria and treated with intravenous colistin as salvage therapy. Colistin may act synergistically with rifampin or carbapenems against metallo-β-lactamase-producing (MBL) *K. pneumoniae *or *A. baumannii *strains. However, these data on colistin are chiefly from nonrandomized studies in small numbers of patients. Although the manufacturer recommends not exceeding 6 million units per day, a growing number of clinicians now routinely use daily dosages of up to 9 million IU in two to four divided doses (12,500 IU of colistin is equivalent to 1 mg of the prodrug colistin methanesulfonate). Inhalational colistin therapy has long been used in patients with cystic fibrosis and is now proposed in critically ill patients with ventilator-associated pneumonia. However, clinical data are derived from small, retrospective, nonrandomized studies. We have very little information on colistin pharmacokinetics and pharmacodynamics, especially in critically ill patients. Recently, Lu et al. reported that inhaled colistin resulted in relative higher lung tissue bioavailability in piglets compared with animals treated with intravenous colistin [35]. Nephrotoxicity associated with the use of colistin remains a matter of concern. In vitro studies have shown that the toxic effects of colistin on mammalian cells are concentration-dependent and time-dependent. Colistin nephrotoxicity seems rare in young patients with normal renal function and common in patients with underlying renal insufficiency. The risk may depend on the cumulative dose. Colistin nephrotoxicity is usually mild and almost always reversible within a few weeks or months after treatment discontinuation.

When no antibiotic-resistant Gram-positive bacteria are isolated, withdrawal of glycopeptides is warranted and may limit the emergence of vancomycin-resistant enterococci and the risk of nephrotoxicity [1,2,11]. Similarly, de-escalation (e.g., switching from carbapenem to cefepime or piperacillin-tazobactam) should be encouraged when no microorganism resistant to the first-line regimen is isolated.

In patients with sustained bacteremia and/or persistent fever and clinical deterioration, a portal of entry requiring specific treatment should be sought. Necrotizing cellulitis or peritonitis requires surgery. One of the commonest problems is deciding whether to remove the indwelling central venous catheter in bacteremic patients. The decision rests on the clinical presentation (septic shock, local tunnel, or port infection), pathogen, presence of intestinal colonization, and differential time to blood culture positivity of samples drawn simultaneously by phlebotomy and through the catheter. In patients with bacteremia due to *Enterobacteriaceae*, enterococci or *Pseudomonas*, with no local signs of catheter infection or septic shock, the conjunction of intestinal colonization and microbial growth in peripheral blood before or within 2 hours after growth in a sample obtained simultaneously from the catheter hub often indicates bacterial translocation from the intestine [36]. Further information regarding the management of catheter-related infections can been found in recently published guidelines [37].

Current guidelines recommend continued intravenous administration of antibiotics after 48 hours of apyrexia, for at least 2 days after neutropenia resolution or for 4-5 days if the fever persists [1]. Clinically or microbiologically documented infection may require longer treatment but with narrower-spectrum antibiotics (i.e., a neutropenic patient who has bacteremia due to multisusceptible *E. coli *treated with piperacillin-tazobactam should be switched to amoxicillin for a few additional days after neutropenia recovery).

International guidelines recommend that patients with persistent neutropenia remain on antibiotics for at least 2 weeks. All patients with persistent fever, with or without clinical deterioration, should be investigated for noninfectious causes of fever (Table 4). Finally, antibiotic dose adjustment based on serum concentration determination may be required, because changes in the pharmacodynamics and pharmacokinetics of antibiotics have been reported in neutropenic patients [38-40].

**Table 4:** Causes of fever persistence after initial empirical antibiotic in neutropenic patients

Infectious causes of persistent fever		
	Inappropriate antibiotic dosing and concentration	
	*Clostridium difficile*-induced diarrhea	
	Antibiotic-resistant pathogen	Multidrug-resistant bacteria, Mycobacteria, Fastidious pathogens (e.g., *Legionella*, *Mycoplasma*, *Chlamydia pneumoniae, Bartonella*)
	Fungal infection	Molds: *Aspergillus *and zygomycetes Yeasts: *Candida *and *Cryptococcus*
	Parasitic infection	e.g., *Toxoplasma gondii*
	Viral infection	e.g., herpesviruses (cytomegalovirus, Epstein-Barr virus, human herpesvirus 6, varicella-zoster virus, herpes simplex virus) parainfluenza virus, respiratory syncytial virus, influenza viruses.
	Persistent focus of infection (e.g., catheter)	
	Uncontrolled infection (e.g., endocarditis or peritonitis)	
**Noninfectious causes of persistent fever**		
	Transfusion-related fever	
	Hemophagocytic lymphohistiocytosis	
	Venous thrombosis	
	Drug- or transfusion-induced fever	
	Graft-versus-host disease	
	Underlying malignancy	
	Pancreatitis	

### Pharmacodynamic and pharmacokinetic considerations

The pharmacokinetics and pharmacodynamics of many antibiotics are modified in neutropenic [38] and/or critically ill patients. The volume of distribution and clearance are increased, and therefore the half-life and plasma concentrations may be lower than in control patients. Many animal studies found decreases in the bactericidal activity of β-lactams in neutropenic animals. Given that the activity of β-lactams and glycopeptides depends on the time spent with serum drug concentrations greater than the minimum inhibitory concentration of the organism, decreasing the interval between doses or using a continuous infusion may be the best strategy for administering β-lactams and glycopeptides to neutropenic patients. In any case, therapeutic drug monitoring is valuable for guiding dosage adjustments and ensuring that therapeutic concentrations are achieved to increase the chances of eradicating the organism and to minimize the risk of selecting antibiotic-resistant bacteria [38,41]. Serum vancomycin and teicoplanin levels should be monitored routinely [39]. In neutropenic patients who receive the recommended 2 g/day dose of imipenem, many may have serum concentrations below the therapeutic range [41]. When using carbapenems in neutropenic patients, 50-60% of the dosing interval must be spent above the minimum inhibitory concentration to achieve bactericidal activity, and success rates improve when 75-100% of the dosing interval is spent above the minimum inhibitory concentration [41-43]. Plasma carbapenem levels can be measured by using high-performance liquid chromatography in patients with persistent fever to ensure that the plasma concentrations are within the therapeutic range.

Aminoglycosides, in contrast, have a concentration-dependent bactericidal activity. Elimination of aminoglycosides is highly dependant on renal clearance, and accumulation is likely to occur in patients with renal failure. Once-daily administration of aminoglycosides often is preferred over multiple-daily dosing to reduce the risk of nephrotoxicity and is recommended in the few neutropenic patients who require an aminoglycoside in combination with a wide-spectrum β-lactam [28]. When using aminoglycosides, therapeutic drug monitoring is important to minimize the risk of renal and cochlear/vestibular toxicity [40]. A recently published review is available for readers who wish further information on antibiotic pharmacokinetics and pharmacodynamics in neutropenic patients [38].

### Prophylactic antibiotics and hygiene mesures

The guidelines issued by the European Conference on Infections in Leukaemia recommend prophylactic ciprofloxacin or levofloxacin therapy from chemotherapy initiation to neutropenia resolution [28]. According to the most recent Infectous Diseases Society of America (IDSA) guidelines, such prophylaxis should only be considered [2]. In fact, many centers still do not give quinolone prophylaxis to patients with afebrile neutropenia, due to concerns about the emergence of antibiotic resistance in the long run [17]. The selection of resistant organisms by quinolones is a serious hazard, as re-emphasized recently [44]. The emergence of resistant strains should be monitored in centers where quinolone-based prophylaxis is used [2,10,45]. Hand hygiene remains the most effective measure to prevent hospital-acquired infections, whereas isolation into a single-patient room or use of specific protective gear (gowns, gloves, and masks) are not mandatory except for hematopoietic stem cell transplant recipients.

## Conclusions

Antibiotic therapy must be initiated promptly in febrile neutropenic patients. In high-risk patients, initial empirical treatment with piperacillin-tazobatam or cefepime is recommended. In addition, the initial antibiotic strategy should be adapted based on the basis of initial clinical assessment, bacterial ecology in the hospital, and bacterial history in the patient. The use of vancomycin should be reserved for patients with suspected methicillin-resistant Gram-positive infections and/or signs of severe sepsis or septic shock. There is some evidence to support adding an aminoglycoside to the extended-spectrum β-lactams in critically ill neutropenic patients. Persistent fever requires adaptation of the initial antibiotic regimen if the clinical condition deteriorates or if a resistant pathogen is isolated. Addition of an antifungal agent must be considered. Giving the growing emergence of multidrug-resistant bacteria, the implementation of antibiotic stewardship programs is now mandatory.

## Competing interests

The authors declare that they have no competing interests.

## Authors' contributions

ML and AM wrote the first draft of the manuscript. BS, EA, and BG revised the manuscript. All authors read and approved the final version.
